# The Search for Cognitive Terminology: An Analysis of Comparative Psychology Journal Titles

**DOI:** 10.3390/bs3010133

**Published:** 2013-02-07

**Authors:** Cynthia Whissell, Charles I. Abramson, Kelsey R. Barber

**Affiliations:** 1 Psychology Department, Laurentian University, Sudbury, Ontario P3E 2C6, Canada; 2 Laboratory of Comparative Psychology and Behavioral Biology, Departments of Psychology and Zoology, Oklahoma State University, Stillwater, OK, 74078, USA

**Keywords:** behaviorism, mentalist/cognitive terminology, titles, emotion

## Abstract

This research examines the employment of cognitive or mentalist words in the titles of articles from three comparative psychology journals (*Journal of Comparative Psychology, International Journal of Comparative Psychology, Journal of Experimental Psychology: Animal Behavior Processes*; 8,572 titles, >100,000 words). The Dictionary of Affect in Language, coupled with a word search of titles, was employed to demonstrate cognitive creep. The use of cognitive terminology increased over time (1940–2010) and the increase was especially notable in comparison to the use of behavioral words, highlighting a progressively cognitivist approach to comparative research. Problems associated with the use of cognitive terminology in this domain include a lack of operationalization and a lack of portability. There were stylistic differences among journals including an increased use of words rated as pleasant and concrete across years for *Journal of Comparative Psychology*, and a greater use of emotionally unpleasant and concrete words in *Journal of Experimental Psychology: Animal Behavior Processes*.

## 1. Introduction

Psychology is currently defined, for those not familiar with it, as “the study of mind and behavior” (by the American Psychological Association [1]) or the “scientific study of behavior and mental processes” (in an introductory psychology text [2]). This bifurcated definition highlights an enduring controversy within the discipline involving the demarcation of its appropriate subject matter. Each of psychology’s two accredited founders dwelt on the importance of mental processes: Wundt favored introspection as an experimental technique while James wrote at length on the topic of consciousness [2]. These approaches were challenged by Watson [3] who repudiated both introspection and consciousness in his flagship article “Psychology as the Behaviorist Views It”. Continuing the challenge, Skinner [4] disallowed any role for mental processes in the *science* of psychology and specifically defined himself as “*not* a cognitive psychologist” [5]. Skinner [4] insisted that mentalist terms not only fail to explain behavior but also interfere with approaches that might explain it successfully. He viewed the two-part definition of psychology as an unworthy compromise reached in an attempt to sell more textbooks [4].

The Stanford Encyclopedia of Philosophy [6] distinguishes behaviorism in terms of three main tenets: that psychology is the scientific study of behavior *not* mind, that external (environmental) rather than internal (mental) causes should be employed to predict behavior, and that mentalist terminology has no place within research and theory (it should be replaced by behaviorist terminology). For radical behaviorists, the two aspects of the current definition of psychology—the mentalist/cognitive and the behavioral—are incompatible. One would not therefore expect mentalist or cognitive terms to be employed with any appreciable frequency in behaviorally oriented efforts. 

The research described in this paper examines the employment of cognitive terms in the titles of articles from three comparative psychology journals: the *Journal of Experimental Psychology: Animal Behavior Processes*, the *Journal of Comparative Psychology*, and the *International Journal of Comparative Psychology*. The study of animal behavior, which concerns these journals, should be relatively free of cognitive terminology.

Titles represent attempts to abstract the sense of an article in order to inform and attract potential readers [7,8,9]. When writers use a certain class of title words more or less frequently, their preferences reflect the status of the approach described by the words. For example, in a study of *American Psychologist* titles, Whissell [9] reported that words from the root “behav” were used three times as often as words from the root “cogni” in early titles (1946–1955: 7 per 10,000 title words *versus* 2) and twice as often in titles from an intermediate period (1979–1988: 43 per 10,000 title words *versus* 22). In recent years both types of words were used less often but not at different rates (2001–2010: 11 and 12 per 10,000 title words). The ratio of cognitive to behavioral words rose across time from 0.33 to 0.50 to 1.00. 

Titles have been studied in terms of their length, their employment of punctuation marks and the emotional connotations underlying their words [7,10,11,12]. In general, psychology titles have become longer over time, have included more punctuation marks, and have increased in terms of their pleasant connotations [9].

## 2. Operational Definitions: Emotional Connotations and Cognitive Words

Skinner [4] suggested that mental processes could serve as data for the behaviorist if they were interpreted as behaviors and clearly defined in terms of operations rather than abstractions. The emotional connotations of journal titles in this research were evaluated using the DAL or Dictionary of Affect in Language [13]. The DAL contains participants’ rated impressions of the connotations underlying many commonly employed English words along three scales—Pleasantness, Activation, and Concreteness. The DAL is composed of several thousand words accompanied by the three ratings. For example, in the DAL the word “action” has scores, which indicate that its connotations are mildly pleasant (z = 0.36), very active (z = 2.67), and quite concrete (z = 1.05), while the word “thought” has scores, which describe its connotations as mildly pleasant (z = 0.36), mildly passive (z = −0.36), and quite abstract (z = −1.17). A behaviorist viewing these scores might affirm that “thoughts,” in contrast to “actions”, could not be easily defined in terms of operations because of their abstract nature: they would therefore not be good candidates for scientific study. An early version of the DAL was employed to examine titles and abstracts of highly cited psychology journals (Whissell, 1999). In this study, the journal *Memory and Cognition *contained some of the most abstract materials studied.

Emotional connotations depend on the rating behaviors of several participants and are interpreted as representing these behaviors. The “pleasantness” or “concreteness” of a word is the rating assigned to it, and the characteristics of an entire text are based on a summation over words. In previous work, the DAL has been used to describe the overall tone of materials and also their structure (tone as a function of time, [9,12,14]). The DAL is a useful operational tool for the study of complex linguistic materials. The inclusion of cognitive terminology within a text is also a behavior: it can be operationally defined (e.g., in Table 1) as involving the presence of particular words in article titles. 

In addition to examining the use of mentalist/cognitive words in titles, the present study considered title length, word length, and the emotional connotations of title words. Based on previous research, it was predicted that titles would grow longer over time, and also more pleasant in tone. The main exploratory question of the study involved the use of cognitive words in the titles of articles in the three journals, and changes in this use over time, especially in comparison to the use of behavioral words.

## 3. Methodology

Titles for the three journals were downloaded from databases. The basic unit of analysis was the volume or year. There were 71 volume-years (1940–2010) for the *Journal of Comparative Psychology*, 11 (2000–2010) for the *International Journal of Comparative Psychology*, and 36 for the *Journal of Experimental Psychology: Animal Behavior Processes* (1975–2010). Hereafter the three journals are referred to in brief as JCP, IJCP, and JEP respectively. A total of 8,572 titles including almost 115,000 words were analyzed. 

Volume-years were scored in terms of the relative frequency of the mentalist or cognitive words that appeared in their titles (Table 1). Cognitive words or phrases were defined as those referring to mental processes (e.g., memory, meta-cognition), emotions (e.g., affect), or presumed brain or mind processes (e.g., executive function, concept formation). Volume-years were also scored in terms of their employment of words from the root “behav” and in terms of their references to vertebrates (e.g., monkey, rodent) and invertebrates (e.g., bees, squid).

**Table 1:** Words and phrases scored as having a mentalist or cognitive emphasis in addition to all words including the root “cogni”.

Words which satisfied the following criteria were counted as cognitive words.		
(1) All words including the root “cogni-”.	(2) Each of the following words or their plural forms (exact matches):	(3) Each of the following phrases (exact matches):
	Affect/s, attention/s, awareness/es, categorization/s, communication/s, cognition/s, concept/s, emotion/s, expectancy/ies, frustration/s, identity/ies, incentive/s, information/s, intelligence/s, imagery/ies, knowledge/s, language/s, logic/s, metacognition/s, metaknowledge/s, memory/ies, mind/s, motivation/s, perception/s, personality/ies, planning, reasoning/s, representation/s, surprise/s, thinking, schema/s.	amodal completion, cognitive development, cognitive maps, concept formation, decision making, declarative learning, executive function, information processing, internal representation, internal states, internal structure, logical reasoning, meta-knowledge, mental images, mental structure, problem solving, procedural learning, selective attention, sequential plans, spatial memory, spatial learning, traveling salesperson ^1^.

^1 ^These words describe a task involving strategy.

The Dictionary of Affect in Language (DAL) was employed to score all title words. A computer program attempted to match every title word to the DAL. When a match was identified, the DAL ratings for that word were added to the data set. Although the DAL has a normative matching rate of 90% (it finds values for most words in everyday English), the matching rate for the titles studied was 69%. Scientific research titles are quite different from everyday English texts because they employ many rare words (e.g., cetaceans, bonobo, inaugurating, exteroceptive), which cannot be scored in terms of their emotional connotations. The emotional results discussed below are based on roughly 79,000 scored words.

Title length was measured in number of words and word length in number of letters. In addition to its source journal and year, each volume-year of titles was described in terms of nine variables: its employment of cognitive terms, its employment of words with the root “behav,” its mention of vertebrate animals, it mention of invertebrates, the Pleasantness, Activation, and Imagery of its words, the length of its words, and the length of its titles.

## 4. Results

According to overall means, titles were 13.40 (SD = 2.34) words long and employed words with 5.78 (0.37) letters. They included cognitive words with a relative frequency of .0105 (0.0077) and words from the root “behav” with a relative frequency of 0.0119 (0.0074). This means that 105 of every 10,000 title words were classified as cognitive and 119 as coming from the root “behav.” There was no significant difference between the usage rate for cognitive and behavioral words (t_117 _= 1.11, p = 0.27). Vertebrates (0.0391, SD = 0.0129) were mentioned much more frequently than invertebrates (0.0012, 0.0022). This difference was statistically significant (t_117 _= 32.39, *p* < 0.001). In comparison to normative English texts such as novels, essays, newspapers, and radio and television broadcasts [15], titles were unpleasant (1.792, t_117 _= −20.59, *p* < −0.001), active (1.732, t_117_ = 25.39, *p* < 0.001) and concrete (1.626, t_117 _= 15.19, *p* < 0.001). 

The three journals (JCP, IJCP, and JEP) were compared to one another in terms of the nine title variables for the 11 years during which all three were in publication (2000–2010). A multivariate analysis of variance indicated strong significant differences among journals (Pillai’s F_18,46 _= 8.46, *p* < 0.001, η^2 ^= 0.78). According to follow-up univariate tests, there were significant differences for all variables except of the mention of invertebrates and the use of cognitive words (Table 2). The strongest effects were associated with word length (η^2^ = 0.85), title length (η^2^ = 0.80), and word Concreteness (η^2^ = 0.60). As indicated by the results of the *post hoc* tests summarized in Table 2, JCP had the shortest words but the longest titles, and it also had the most concrete titles. In contrast, JEP had the most abstract and the shortest titles which included relatively long words. JEP titles were the least pleasant and the least active. In terms of its greater use of “behav” words, IJCP was more behaviorally oriented than the other two journals. In the 11 recent years tested, IJCP used cognitive words at the same rate as “behav” words, while the remaining two journals used significantly more cognitive than “behav” words (*t *tests, *p *= 0.008 for JCP, 0.996 for IJCP, and 0.001 for JEP): JCP employed two times as many cognitive as “behav” words and JEP four times as many (Table 2). 

**Table 2:** Means and differences among means for the three journals on the nine title variables (2000–2010)^1,2^.

Title	Journal					
Variable	JCP	IJCP	JEP	F_2.30_	*p*	*η^2^*
DAL Pleasantness	1.829 ^a^	1.818 ^a^	1.785 ^b^	7.23	0.003	0.325
DAL Activation	1.734 ^a^	1.742 ^a^	1.704 ^b^	5.54	0.009	0.270
DAL Concreteness	1.701 ^a^	1.640 ^b^	1.563 ^c^	30.49	< 0.001	0.670
Word Length	5.276 ^a^	5.998 ^b^	6.175 ^c^	86.08	< 0.001	0.852
Word Number	17.787 ^a^	13.025 ^b^	12.611 ^b^	60.56	< 0.001	0.901
Root “Behav” Words	0.007 ^a^	0.015 ^b^	0.004 ^a^	12.79	< 0.001	0.460
Cognitive Words	0.014	0.015	0.017	0.28	0.755	0.019
Vertebrate Words	0.047 ^a^	0.034 ^b^	0.026 ^b^	9.61	0.001	0.390
Invertebrate Words	0.003	0.003	0.000	2.28	0.119	0.132

^1 ^JCP: Journal of Comparative Psychology; IJCP: International Journal of Comparative Psychology; JEP: Journal of Experimental Psychology: Animal Behavior Processes;^ 2 ^Any two means in the same row with different superscripts (a, b, or c) are significantly different according to Student-Newman-Keuls *post hoc* tests.

Because of the mismatch in years, title variables were correlated with year for each of the three journals separately. There were no significant changes across time for JEP or IJCP, but several trends were evident for JCP. It was confirmed that lack of significance was not merely due to lack of power for the smaller samples, where correlation coefficients were actually smaller. Titles in JCP grew longer with time (r = 0.86), but they employed shorter words (−0.42). Pleasantness (0.74) and concreteness (0.80) of words rose across time, and both vertebrates (0.25) and invertebrates (0.26) were mentioned somewhat more frequently. The relative frequency of “behav” words decreased across years (−0.49) while the relative frequency of cognitive words increased (0.69). Results involving title length, pleasantness, and concreteness are in accord with previous research [9,12]. The drop in use of behavioral words and the rise in cognitive words also match previous findings [9], although the two categories of words were not operationalized in the same way.

Cognitive creep is reflected in the increased use of cognitive words in comparison to behavioral words across time. For all 118 volume-years studied, the correlation between the difference of these two variables (the use of cognitive—the use of “behav” words) with year was 0.59 (*p* < 0.001). In the first 20 years (1946–1965), the difference was in favor of the use of behavioral words (−0.010) and in the last 20 (1991–2010) in favor of the use of cognitive words (0.006). The difference of differences is significant (*t* test, *p* < 0.001). 

**Figure 1:**
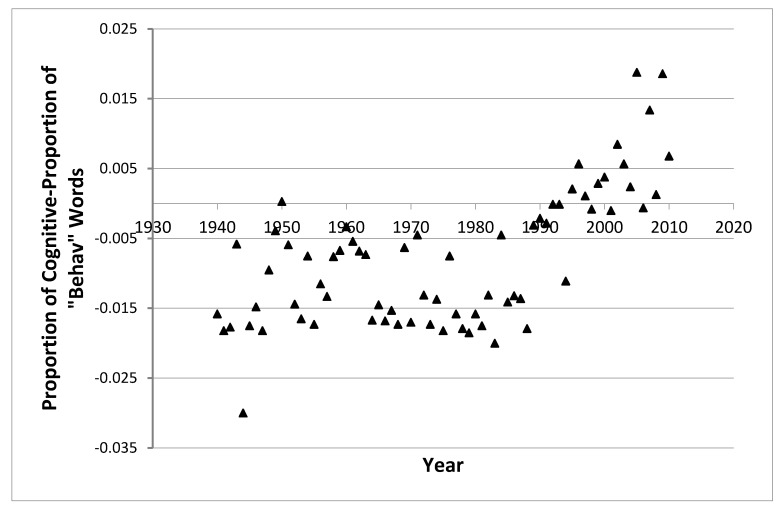
Cognitive creep: a relative increase in the employment of cognitive terminology over time for JCP.

For JCP alone the relationship of cognitive creep with year, which is shown in Figure 1, was quadratic (r_q _= 0.75). A line drawn through the center of the data points would change in direction. As is evident in Figure 1, cognitive creep does not seem to take hold until the 1980s, after which it proceeds relentlessly. In the 1990s the employment of cognitive words actually exceeds that of “behav” words. This is where the points in Figure 1 appear above the zero line where the use for two types of words is equal. For the years between 1940 and 1980, the correlation between the difference and year in JCP was insignificant (−0.03, p > 0.10), and for the years between 1991 and 2010, it was direct, significant and strong (0.84, p < 0.01). Unless a second change in direction can logically be predicted, these data suggest that cognitive creep will continue. The first change in direction is likely mirroring the upswing of cognitive psychology (which included the founding of several journals) in the 1970s.

## 5. Discussions

Committed behaviorists have taken something resembling a vow of celibacy: they are determined to have no intercourse whatsoever with mentalist terminologies [5]. This type of thinking can be traced to the strong influence of logical positivism (represented in the work of authors such as Carnap and Ayre) on early 20^th^ century thinking, and especially on behaviorist thinking. The aspect of logical positivism embraced by behaviorists is the one focusing on empirical evidence (evidence from the senses) and operationalization (the definition of variables in terms of the actions taken in measuring them). Cognitive terminologies, which often refer to inferred constructs, do not rely directly on empirical evidence. Some of them cannot be operationalized, even with the best will in the world. These are the exact criticisms leveled by Carnap [16] at the philosopher Heidegger whose metaphysical writings were awash in indefinable words. 

We cannot assess the minds of others empirically nor can we satisfactorily operationalize variables such as “consciousness,” “memory”, and “cognition”. That is why Skinner, who was willing to admit that there *might* be such a thing as “mind”, insisted that psychologists should place it in a black box and limit their research efforts to cue stimuli, reinforcing stimuli, and behaviors [5]. Behaviorists do not deny the existence of “mind”, merely its usefulness in psychological research. If one asked a behaviorist “What is mind?” her or his answer would likely be “No matter”, which one could accurately interpret either as “It doesn’t matter”, or as “The mind immaterial and cannot be studied.”

If one were to take only a few puffs of cognitive terminology now and then, always forbearing to inhale, what damage could it do? According to behaviorists, cognitive terminology is not only use-*less*, but it is harm-*full*. Problems that arise when psychologists talk about indefinable entities include confusion, misdirection, and the fatal obfuscation of fact. Rogue metaphors and poorly understood terminologies provide two examples of these problems.

It is possible to argue that there are benefits to the metaphorical employment of cognitive terminology. For example, students studying memory for the first time in introductory psychology courses might find the Atkinson-Shiffrin three-box model, which includes sensory, short-term, and long-term memory ([2], p. 242), useful in helping them understand that not all things are remembered in the same way. The danger of using un-operationalized metaphors such as this one lies in the possibility that the metaphors might go rogue and hijack the research. Impressionable first year students offered the three-box model as an explanation of memory later become researchers who argue endlessly about whether recall tested 10 minutes after exposure to a list of words is accessed from short-term memory, long-term memory, some combination of the two, or “none of the above”. One wonders whether these budding cognitivists are actually envisioning boxes in people’s heads—with arrows leading from one box to the other. A sound behaviorist would advise researchers to drop all references to memory and stick to describing the conditions of the experiment—*i.e.*, the 10 minute lapse between exposure to material and testing. The behaviorist would also stress that the words written down by a participant at the end of 10 minutes would reflect recall (an operationalized behavior) not memory (a cognitive concept).

“Attention” is a culpable cognitive term. It has been studied for many years, and several tests have been created to measure it. A graduate student at Laurentian University (B. Wroblewski) studied measures of attention. She collected several tests claiming to measure attention, several others claiming to assess inhibition, and some measures of scholastic performance, and administered them to a large group of elementary school children. Wroblewski was searching for convergent and discriminant validity for the measures of attention. Her hope was that attention measures would correlate strongly with one another but not with other types of measures. In fact, rank correlations among measures of attention were mainly insignificant, though sometimes extremely weak (below 0.25). The same measures of attention correlated in a similar manner with measures of inhibition (a different cognitive concept) and scholastic performance. There was nothing in Wroblewski’s data that would allow her to point to a test with assurance and say “This test measures attention, the whole of attention, and nothing but attention.” There was some degree of operationalization in the each of the many tests created to measure attention, but the use of the word itself remains problematic for two main reasons. The first reason is that not all the tests claiming to measure attention were measuring the same thing—their results were not related to one another. The second reason is that researchers continue to talk about attention as if it were an actual (and uniform) entity, without satisfactorily defining it. This is called reification. Many studies of attention in school children have been used to inform teachers, parents, and even governments forming policy about how to treat children. These audiences assume that when psychologists talk about attention they know what they are talking about, which is a dangerous assumption. When one person means one thing by “attention” and another means another, the word does more harm than good in the scientific vocabulary.

Since the middle of the 20^th^ century, there has been an upswing in the employment of cognitive terminology within a variety of psychological domains [17]. Some interpret this upswing as a return to normalcy after a behaviorist “rupture” in the dominant mentalist tradition of the discipline [18], while others note the presence of concurrent upswings in the areas of neuroscience and cognitive neuroscience which seem to hold promise as the future of psychology [19]. In reference to the up-and-coming field of cognitive neuroscience Turner [20] affirmed that mentalist terms such as “attention” and “volition” are not portable across cultures, and warned that “it is easy to overestimate the generality of mentalistic constructs” ([20], p. 31). Turner did not suggest abandoning such constructs, but rather defining them more carefully. The data described in this paper address the use of cognitive words in research focusing on animal behavior. What meaning can cognitive words have when they are applied to animals whose “minds” researchers cannot access in any direct fashion? With animal participants even the mediation of language, commonly employed to validate mentalistic terms in reference to human participants, is denied to the researcher. 

## 6. Conclusions

There is a tendency for cognitive words to appear with increasing frequency, and at a greater rate than behavioral words, in the titles of articles from comparative psychology journals. This tendency is associated with moderately strong effect sizes (r^2^) ranging from 0.24 to 0.56. The trend is most obvious in JCP (Figure 1) and absent from IJCP which did not employ more cognitive than behavioral words only because it employed so many of both (Table 2). Increases in cognitive terminology for JCP began during the 1980’s. Titles from JCP increased in length, pleasantness, and concreteness over time. They also included more mentions of both vertebrate and invertebrate animals. The increasing use of cognitive words in titles, labeled as cognitive creep, is an issue because readers of the research literature have no assurance that the meaning in such words is portable across research situations. The operationalization of behavioral concepts permits for greater portability. 

The Dictionary of Affect in Language coupled with a word search of titles was employed to highlight cognitive creep in the titles of journals dealing with comparative psychology and animal behavior. These techniques can be applied in similar ways to other texts (e.g., introductory texts; different journals). It would interesting to analyze how behaviorism is portrayed in cognitive and learning texts respectively. Our results suggest that it would not be surprising that even in learning texts there is a significant amount of “cognitive creep.” The DAL is available [21] for research purposes and can be applied relatively easily by student researchers analyzing textbooks. The word substitution commands within processing programs such as Microsoft Word® can also be employed to search for selected terms.
